# Occlusion and Biomechanical Risk Factors in Implant-Supported Full-Arch Fixed Dental Prostheses—Narrative Review

**DOI:** 10.3390/jpm15020065

**Published:** 2025-02-07

**Authors:** Andrea Berzaghi, Tiziano Testori, Riccardo Scaini, Sergio Bortolini

**Affiliations:** 1 Department of Surgery, Medicine, Dentistry and Morphological Sciences with Interest in Transplant, Oncology and Regenerative Medicine, University of Modena and Reggio Emilia (UNIMORE), 41125 Modena, Italy; sergio.bortolini@unimore.it; 2 IRCCS Galeazzi S. Ambrogio Hospital, Dental Clinic, Section of Implant Dentistry and Oral Rehabilitation, 20157 Milan, Italy; tiziano.testori@unimi.it (T.T.); riccardoscaini@icloud.com (R.S.); 3 Department of Biomedical, Surgical and Dental Sciences, University of Milan, 20122 Milan, Italy; 4 Department of Periodontics and Oral Medicine, School of Dentistry, University of Michigan, Ann Arbor, MI 48109, USA; 5 Department of Oral Medicine, Infection and Immunity, School of Dental Medicine, Harvard University, Boston, MA 02115, USA

**Keywords:** implant-supported full-arch fixed dental prosthesis, occlusion, occlusal scheme, occlusal overload, occlusal materials, personalized occlusion, biomechanical risk factors

## Abstract

The biophysiological differences between teeth and dental implants and the issue of occlusal overload, although controversial, form the basis for the management of occlusion in implant-supported full-arch fixed dental prostheses (ISFAFDPs). Although there is currently a lack of scientific evidence on occlusal management, it is clear that the favorable prognosis of ISFAFDPs is linked to a correct understanding of the biomechanical principles involved. In the design of ISFAFDPs, the lack of proprioceptive feedback requires special attention to biomechanical factors: minimizing overloading complications and providing biomechanical stability are among the main goals of the occlusion. In ISFAFDPs, the occlusion must be decided on the basis of several factors that influence the loads on prosthesis and implants: each case must be evaluated individually and requires a personalized occlusion. The main aim of this narrative review is to provide an overview of the occlusal principles and materials that can be used in ISFAFDPs based on the data currently available in the literature. Practical clinical recommendations for the occlusion management of ISFAFDPs and a biomechanical risk score index to personalize implant-prosthetic treatment are proposed.

## 1. Introduction

Edentulism is debilitating and irreversible and has been described as the ‘ultimate marker of oral health disease burden’ [1]. It is also associated with a decrease in patients’ quality of life, loss of self-esteem and potential impact on patients’ life expectancy [2,3,4]. For fully edentulous patients, the treatment options available are different and may include removable denture with and without implants and fixed prosthesis on implants. Implant-supported full-arch fixed dental prostheses (ISFAFDPs) represent the gold standard of care and aim to restore the aesthetics and function to edentulous patients, enabling them to improve their oral health and return to a normal lifestyle [3,4,5]. ISFAFDP can now be considered a routine procedure in implant dentistry. Advances in imaging techniques, virtual surgical planning, implant designs, prosthetic connections, prosthetic technologies and fabrication materials have all contributed to the evolution of this complex prosthetic solution. ISFAFDPs can be fabricated with a variety of prosthetic designs in a wide range of material combinations selected based on clinical and economic factors. Retention mode, framework design and combination of prosthetic materials are the main differences between the available treatment options. The recent evolution of CAD/CAM technologies together with the introduction of the latest generation of prosthetic materials with high mechanical and aesthetic performance has brought undeniable advantages, simplifying the execution of these rehabilitations from both the design and manufacturing points of view.

Several studies have documented a favorable long-term prognosis with high implant survival rates [6,7]. Several reports have shown that full-mouth implant reconstructions are successful [8,9].

However, ISFAFDPs may present prosthetic and biological complications due to several variables including occlusion: occlusal design, occlusal contact distribution and number, framework design, the type of antagonist arch and the presence of parafunctions are among the variables that may determine the biomechanical stability of implant-supported rehabilitation [10,11,12,13]. The issue of occlusion in ISFAFDPs is as debated as it is complex, involving surgical and prosthetic aspects that require careful planning and design. In ISFAFDPs, decisions about prosthetic occlusion include the choice of materials, the selection of implant–prosthetic design, and the definition of the distribution and intensity of occlusal contacts. The difference in biomechanics between teeth and implants makes occlusal design complex: achieving proper occlusion requires an understanding of the biophysiological differences between natural teeth and implants, as well as recognizing the potential role of occlusal overload in the development of prosthetic and biological complications. These concepts form the basis for the occlusal strategies in ISFAFDPs and for the selection of appropriate occlusal materials. A wide range of combinations of prosthetic materials are available to clinicians for the realization of ISFAFDPs, selected on the basis of clinical criteria and economic availability to the patient. Understanding the strengths and weaknesses of the available options is critical to selecting the most advantageous implant-supported solution. Knowledge of occlusal concepts, occlusal materials and occlusion-related risk factors influences the long-term prognosis of implant-supported restorations [14]. The aim of this study is to provide an up-to-date overview of the occlusal principles and materials that can be used in ISFAFDPs based on currently available data in the literature. It proposes practical clinical recommendations for the management of occlusion in ISFAFDPs and a biomechanical risk score index aimed at personalizing implant-supported treatment.

## 2. Materials and Methods

An electronic search was conducted using PubMed/MEDLINE on 30 June 2024. The following keywords were used: ‘occlusion’ AND ‘dental’ AND ‘implant’. Filters applied: from 30 June 2004 to 30 June 2024 (last 20 years). No limit was set. The electronic PubMed search yielded 2002 results. The inclusion criteria focused on articles, reviews and clinical trials that addressed the management of occlusion in ISFAFDPs, with particular reference to occlusion guidelines, occlusal schemes and occlusal concepts. The in vitro and finite element analysis studies were included because they complemented the occlusion analysis using different methodologies and allowed for better comprehension of the biomechanical principles involved. The exclusion criteria filtered out single or partial implant rehabilitation, case reports and animal studies. Only English language articles were included. Titles and abstracts were selected for inclusion, and eligibility was assessed by reading the full text. A total of 43 papers were identified as potentially relevant, and 15 were selected.

## 3. Results

Evidence-based consensus on the management of occlusion in ISFAFDPs is limited. There are no data from long-term clinical trials to establish an occlusal guideline or specific occlusal scheme to optimize outcomes in ISFAFDPs. The differences between natural teeth and implants and the potential role of occlusal overload, although still controversial, form the basis of occlusal management in ISFAFDPs. Recommendations and suggestions for occlusion in ISFAFDPs are primarily based on expert opinion and intuitive guidelines without a high level of evidence. In this regard, simulation studies using finite element analysis are proving useful in the management of occlusion in ISFAFDPs. Due to the lack of high-level evidence on these topics, a narrative review is provided with practical clinical recommendations and considerations for the management of occlusion in ISFAFDPs.

### 3.1. Differences Between Teeth and Dental Implants

The biophysiological differences between a natural tooth and a dental implant are well established [11]. The structural and functional connection between bone and the titanium implant surface is known as osseointegration and provides a stable biological basis to support the prosthetic restoration [15]. In contrast to natural teeth, osseointegrated implants are anchored in the bone without the presence of the periodontal ligament apparatus, which biologically provides mechanoreceptors and functional shock absorption. This results in significant differences between teeth and implants in terms of load perception and modulation mechanisms and in terms of stress distribution, with implants showing reduced resilience and load-bearing capacity. Teeth have a high degree of proprioception, with the ability to perceive occlusal thickness quantifiable at around 20 μm [11,12,16]. In contrast, this is less for implants: the perception capacity between a tooth and an implant is about 48 μm; between two implants, it is about 64 μm; and for implant-supported overdentures, it increases to about 108 μm [11,12,16]. In addition, implants and teeth react differently under load. In the tooth, the periodontal ligament acts as a shock absorber under load due to its viscoelastic properties, allowing axial movement of approximately 25–100 μm and horizontal movement of approximately 50–100 μm. Loading on the implant, due to the viscoelastic properties of the bone, can induce axial movement of approximately 3–5 μm, while horizontal implant movement can reach 10–50 μm [11,12]. The implant reacts to the load to the extent allowed by the elastic deformation of the alveolar bone. Indicatively, a light force (20 N) can intrude upon a natural tooth by approximately 50 μm compared to approximately 2 μm in the osseointegrated implant [17]. In the implant, horizontal forces are concentrated more at the level of the peri-implant crestal bone, opposing rotation [11]. In ISFAFDPs, the lack of shock-absorbing function due to the compressibility of the periodontal ligament of the natural tooth results in significant differences in adaptation to occlusal forces. Furthermore, while natural teeth have neuromuscular and proprioceptive mechanisms to protect themselves from damaging forces, there is no equally effective mechanism for implant-supported prostheses. The periodontal ligament of natural teeth is able to provide the central nervous system with feedback for sensory and motor control, unlike implants, which only have feedback from distant mechanoreceptors, resulting in reduced tactile sensitivity [16,18,19]. Natural teeth have periodontal mechanoreceptors and proprioceptive and neuromuscular mechanisms that can provide feedback for sensory and motor control to protect against occlusal overload. For normal control of contact and masticatory forces, adequate sensory information from periodontal mechanoreceptors is essential [20]. In ISFAFDPs, periodontal ligament mechanoreceptors and the associated proprioceptive input are completely absent. The reduced tactile sensitivity of the implants may result in reduced coordination of the masticatory muscles and increased susceptibility to occlusal overload [21]. In the absence of a periodontal ligament, the occlusal loads of the implant-supported prosthesis are transferred directly to the bone, which, according to some authors, predisposes to biological complications [19]. Despite these differences between teeth and implants, several studies support the existence of a compensatory phenomenon called ‘osseoperception’, which is the mechanism underlying the functional integration of the dental implant [22]. The term osseoperception has become widely associated with occlusion in cases of implant-supported restorations and indicates that biological compensation process capable of restoring sensory feedback after implant-supported oral rehabilitation. The functional integration of ISFAFDPs is based on osseoperception, which can be defined as physiological compensatory adaptation promoted by mechanical stimulation of the implants. With the loss of teeth and periodontal structure, we see the loss of mechanoreceptors and proprioceptive and neuromuscular mechanisms capable of providing feedback for sensory and motor control to protect against occlusal overload. Nevertheless, other receptors, both proximal and peripheral, seem to take over and are responsible for a physiological compensatory adaptation promoted by the mechanical stimulation of the implant that approaches natural sensitivity [23]: the mechanoreceptive function seems to be evoked by receptors in the osseous, periosteal and peri-implant periodontal tissues and by peripheral receptors at the level of the masticatory muscles and the temporomandibular joint. These provide mechanosensory information regarding jaw function and occlusal contacts of implant restorations. The tactile sensitivity provided by the osseoperception mechanism, although not identical to the original, appears to be qualitatively and quantitatively sufficient to ensure the ability to adapt to occlusal loading and modulate motor activity, even in the peri-implant region [22]. In addition, the perception of occlusal contact with implants is likely to improve over time due to the biological and psychological plasticity of the somatosensory cortex [23,24]. One study reported a significant improvement in osseoperception of implants after 3 months of healing, supporting the compensatory role of other receptors [24]. However, it is unclear whether the level of perception of implants completely returns to that of natural teeth over time [25]. Failure to achieve optimal osseoperception may expose to overload and complications related to excessive occlusal forces that the patient cannot properly perceive in the absence of restored neurosensory function [22]. In this clinical scenario, the prosthetic occlusion must be designed to preserve the ISFAFDPs from excessive occlusal loads potentially favored by loss of proprioception (Table 1).

### 3.2. Occlusal Overload and Dental Implants

Knowledge regarding the relationship between peri-implant disease, dental implant survival rate and occlusal overload is currently limited and lacking in scientific evidence [25]. Occlusion is a known contributing factor to the occurrence of prosthetic complications. However, some authors believe that occlusion also plays a role in biological complications of dental implants. The most common biological complications in implant-supported restorations are peri-implant diseases, particularly mucositis and peri-implantitis [26]. Although the success rates of implant-supported restorations are high, some authors identify peri-implantitis and occlusal overload due to improper occlusion as the main causes of late implant failure [27]. Peri-implantitis is defined as ‘a plaque-associated pathological condition occurring in tissues around dental implants, characterized by inflammation in the peri-implant mucosa and subsequent progressive loss of supporting bone’ [28]. The current literature has attempted to identify factors that may increase a site’s susceptibility to peri-implantitis by identifying five factors in implant design, implant site, prosthesis, and operator- and patient-related variables that may have a synergistic effect on the overall host response to bacterial plaque at implant sites [29]. In addition, it appears that some peri-implant inflammatory conditions may be related not only to biofilm-mediated infectious processes but also to other biological mechanisms, such as the foreign body response [30]. Some authors consider the presence of wear facets on implant-supported rehabilitations as a risk indicator for peri-implantitis [31]. Occlusal overload is the application of excessive force to an implant, either by normal function or parafunction, resulting in structural or biological damage [32]. The absence of a periodontal ligament can make dental implants more vulnerable to occlusal overload: lacking periodontal receptors, they have a reduced capacity for load sharing, adaptation to occlusal forces and mechanoreception, making them more susceptible to overload [11]. Furthermore, implant-supported restorations lack the shock-absorbing function provided by the compressibility and deformability of the periodontal ligament of the natural tooth. According to proponents of a cause–effect link between overloading and biological complications, the presence of peri-implant bone loss is attributable to the presence of occlusal overload. Excessive occlusal loading on implant-supported restorations results in increased stress on prosthetic components and peri-implant bone tissue, particularly in the marginal bone ridge region [11]. This mechanism represents a potential cause of prosthetic damage or peri-implant bone loss and is therefore unacceptable for both technical and biological reasons [33,34]. Peri-implant bone loss is complex, with the bacterial component relevant but often associated with other factors [35]. In addition, local and individual factors can influence the strength of osseointegration, and therefore, the biological effect of occlusal loading is highly variable [36]. Several studies have been conducted to evaluate the impact of overload on peri-implant tissues. However, the link between overload and peri-implant tissue loss remains controversial due to the limited number of clinical studies and the difficulties in conducting randomized controlled trials in humans [29,37,38]. Most of the data on the relationship between occlusal overload and peri-implant bone loss comes from animal studies, with their limitations and conflicting results. While occlusal overload appears to play a role in the development of peri-implant disease in the presence of inflammation, this association is not observed in cases where the peri-implant tissue is clinically healthy [38,39,40,41]. Although the data are conflicting, potential deleterious effects of occlusal loads on peri-implant crestal bone cannot be excluded [35]: occlusal overload can reasonably be considered a predisposing factor for peri-implant disease in the presence of plaque and inflammation [29,38]. Occlusal forces on implant restorations are dynamic and multidirectional [42] and can be influenced by individual factors [43,44]. Some authors argue that the intensity, frequency and duration of occlusal loading can lead to pathological overloads that exceed physiological tolerance of the bone, resulting in microfractures at the bone–implant interface [19]. In addition, the loading intensity may influence the bone response: Frost’s mechanotactic theory [45] considers loading as an agent capable of producing different effects on bone depending on the level of micro deformation produced: micro deformation values above 3000 micro strain (με) are considered to be overload, resulting in a catabolic bone response. Melsen and Lang, on the other hand, observed that bone resorption occurs above 6700 με [46]. The issue of the load threshold value capable of triggering peri-implant bone loss remains complex; still theoretical, without definitive answers; and difficult to relate to clinical reality [47]. Despite conflicting opinions on the cause-and-effect relationship between overload and peri-implant disease, there is evidence that occlusal overload is one of the main causative factors of technical and mechanical complications affecting the prosthesis and supporting implants [38]. In addition to causing fracture of the occlusal materials of the restoration and structural failure of the prosthesis, overload can induce loosening and fracture of the connection screw and also implant fracture [14,38]. Implant fracture is the seemingly rarer but most feared complication. Over time, function can compromise implant integrity by inducing ‘cracks’ that can accelerate or trigger peri-implant bone loss, resulting in exposure of the implant neck and coils [21]. In this regard, a study of implants that failed due to peri-implantitis found a particularly high percentage of implant fatigue cracks [48]. These data confirm that the relationship between overloading, mechanical complications and biological complications is still poorly defined and that this topic requires dedicated studies. Despite the ambiguity of the relationship between occlusal overload and peri-implant bone loss, the occurrence of both technical and mechanical prosthetic complications in ISFAFDPs should be noted and not underestimated, as they may signal more important consequences: they may represent clinical signs predictive of potential biological complications. Frequent loosening or fracture of connection screws as well as peri-implant bone loss are characteristic signs that may precede implant fracture [49]. It is important to note that complications are often interrelated. As stated in a recent review, there is a bidirectional positive feedback between biological and prosthetic complications, meaning that prosthetic complications can lead to biological complications and vice versa [50]. There are numerous factors and clinical scenarios that may contribute to occlusal overload and have a negative impact on the long-term prognosis of ISFAFDPs: inappropriate occlusal scheme or occlusal design, premature contacts, unbalanced static and dynamic occlusal contacts, inadequate passive fit of the prosthetic framework, long cantilevers and parafunctional habits. In addition, other biomechanical variables not strictly related to occlusion may influence the distribution of masticatory forces on the implant: bone quality and quantity; implant length, implant diameter and its macro and micro topography; number and position of fixtures; type of prosthesis; prosthetic material; and type of implant connection [51]. Regarding the number of implants in ISFAFDPs, 6–8 implants in the maxillary upper jaw and 4–8 mandibular implants are considered acceptable [12]. In an FEA study, the all-on-six approach in the edentulous maxilla showed the most favorable biomechanical behavior compared to the all-on-four approach [52]. Regarding bone quality, this should be considered a critical factor in the success of implant treatment. A 20-year retrospective clinical study found that implants placed in type I bone demonstrate the lowest failure rate compared to other bone types [53]. Several studies have reported that the failure of implants in the posterior maxilla is related to the bone quality in that area [54]. In addition, the combination of occlusal overload and poor bone quality has been considered a relevant factor in late implant failure [55]. The results of recent systematic reviews indicate that reducing occlusal overload contributes to favorable prosthesis and implant prognosis [14]. It is suggested that implant–prosthetic occlusion should be optimized in terms of load distribution and occlusal stability to minimize complications and ensure long-term biomechanical stability of the restoration [27,38] (Table 2).

### 3.3. Bruxism

Bruxism has been defined as a common parafunctional activity involving repetitive movement characterized by clenching, grinding, and jaw creeping or thrusting. It has a multifactorial etiology with physiological, psychosocial and external associated factors. Bruxism is not a pathology but should be considered as a behavior that comprises two distinct manifestations: sleep bruxism (SB), which occurs during sleep, and awake bruxism (AB), which occurs during wakefulness [56]. The risk factors for bruxism are controversial and poorly understood. The prevalence of bruxism in the general population ranges from 8 to 31.4% and is particularly common in people under the age of 40, especially in women [56]. Possible consequences of bruxism include increased activity and hypertrophy of the masticatory muscles, increased tooth mobility, dental tissue damage and repeated damage to prosthetic restorations.

Bruxism is a parafunctional activity that increases the risk of prosthetic and biologic complications in implant-supported restorations. Depending on the area of the oral cavity and patient characteristics, occlusal forces vary considerably. The magnitude of occlusal loads varies from 100–250 N at the level of anterior teeth to 300–800 N in posterior sectors. The highest load levels are generally attributable to parafunctional habits with maximum values even exceeding 800 N at the level of the first molar [43,57]. In addition to generating higher forces, bruxers also outperform non-bruxers in terms of contact frequency [19,58,59]. In cases of ISFAFDPs, the lack of proprioception typical of the absence of the periodontal ligament may amplify parafunctional activity [19]. In extensive prosthetic rehabilitations, bruxism may contribute significantly to implant fractures, peri-implant marginal bone loss and subsequent implant failure [60,61,62]. Between 20% and 35.9% of patients may generate forces high enough to cause microfractures of the bone around the implants, resulting in bone loss and implant failure [19]. A study of bruxers and non-bruxers reported an association between bruxism and implant failure with an odds ratio of 2.71 [63]. In addition, a recent study reported implant survival rates in bruxers at 5 years: 90% at 1 year, 87% at 2 years, 85% at 3 years, 75% at 4 years and 72% at 5 years [64]. In another study on implant fracture, 90% of implant fractures occurred in parafunctional patients and in cantilever prostheses [49]. Despite inconclusive evidence, these studies require the clinician to preserve the implant–prosthesis from bruxism-induced loads. Devices such as occlusal splints are commonly used to protect both natural teeth and implant restorations from potential damage caused by bruxism [38]. Although the use of occlusal splints in the treatment of nocturnal bruxism is not supported by scientific evidence and their effectiveness in decreasing nocturnal muscle activity has not yet been proven, these devices help to distribute the occlusal forces evenly, prevent unfavorable loads, and preserve the occlusal material of the restoration from fracture and wear mechanisms [60,65,66].

### 3.4. Cantilever

The distal cantilever, which has a long history of clinical success in ISFAFDPs, is one of the prosthetic components with the highest risk of mechanical complications in cases of limited prosthetic space or parafunctional habits [67,68]. A recent study confirmed that the presence of a cantilever is associated with increased prosthetic complications in zirconia and titanium ceramic ISFAFDPs [69]. In addition, the cantilever is a design component that can generate occlusal overload and stresses on implants, especially on those closest to the extension [19,70]. The cantilever is a source of biomechanical stress: it acts as a class I lever and subjects the implants, implant–prosthetic connection and peri-implant bone to alternating tensile and compressive stresses during function [71]. Increasing cantilever length results in exponential growth in stress levels: some authors consider excessively long cantilevers to cause peri-implant bone loss and prosthetic failure [72,73,74]. The length of the cantilever should not exceed 15 mm in the mandible and 12 mm in the maxilla [11,70,75]. However, to avoid potential complications, some authors consider it prudent to design cantilevers no longer than 8 mm [76]. However, there are factors that may influence the choice of cantilever length. The presence of more splinted implants may allow for an increase in cantilever length [77,78]. Conversely, fewer implants result in more unfavorable bending forces [73]. According to some authors, proper implant placement, shorter cantilevers and the use of long implants may be effective strategies to prevent complications [10]. In ISFAFDPs with cantilevers, the presence of distal tilted implants has demonstrated better stress distribution on the posterior implants than rehabilitations with straight implants [79]. In all-on-four zirconia solutions, shortening the cantilever and increasing the tilt of the posterior implants to 30° appears to be advantageous in terms of load distribution [80]. A recent FEM study suggests a 9 mm cantilever in the presence of distal tilted implants in cases of mandibular monolithic zirconia rehabilitations [81]. Historically, the A-P spread has often been used to determine the cantilever length in ISFAFDPs. The A-P spread is defined as the distance between the center of the most anterior implant and a line joining the distal margins of the two posterior implants: the distal cantilever should remain in a 1.5:1 ratio with the A-P spread. However, the A-P spread method has not been scientifically validated and is only one aspect to consider when determining cantilever length [82]. According to several authors, the cantilever should be designed with a sub-occlusion (clearance) of approximately 100 μm to prevent unfavorable loading and fracture risk [11,17,83]. In addition, there should be no contact on the working and balancing sides of the cantilever during lateral excursion: lateral and protrusive excursion should cause cantilever disocclusion [11,17]. Although ISFAFDPs generally show higher rates of complications when antagonized with natural teeth or fixed restorations [13], some authors suggest that even higher stresses may occur when cantilevers are antagonized with removable total dentures [17]. There are numerous studies on posterior cantilever, but few data are available on anterior cantilever. In edentulism, inter-arch relationships are often compromised by the dynamics of bone resorption, forcing an implant–prosthetic design with an anterior cantilever and vestibular over contour to compensate for the sagittal discrepancy between the mandible and upper jaw. The maxilla is usually more exposed to this prosthetic design due to the need to position the incisal margins according to esthetic, phonetic and occlusal criteria. In the presence of an anterior cantilever, a greater antero-posterior distance between the more distal and more anterior implants may be advantageous to compensate for increased excursion loads. Brosky’s study of mandibular implant-supported rehabilitations with anterior cantilevers ranging from 5.9 to 14.4 mm found no significant correlations between anterior cantilever extension and peri-implant bone loss [71].

### 3.5. Occlusal Materials

The first occlusal material introduced for ISFAFDPs was acrylic resin: according to Branemark’s original protocol, ISFAFDPs consisted of cast gold frameworks combined with acrylic resin and acrylic teeth. Later, the cost of gold led to the use of alternative alloys such as silver–palladium, titanium or chromium–cobalt. In the past, acrylic resin was thought to provide a ‘shock-absorbing’ effect on implants that could compensate for the resilience of the periodontium and allow the occlusal surface to be the weakest link in the implant–prosthetic restoration. The aim was to provide a shock-absorbing effect to reduce overload and the likelihood of implant failure [84]. However, this ‘shock protection concept’ belonged to the early design concepts of ISFAFDPs. Over the years, aesthetic demands and other priorities have led to the introduction of alternative materials. As knowledge of osseointegration and the evolution of materials and implant–prosthetic design has deepened, the use of metal–ceramic systems has become widespread [85]. The combinations metal–acrylic resin, metal–composite resin (in this case, the prosthetic teeth are the composite) and metal–ceramic represent the most traditionally used materials in ISFAFDPs. The combination of metal framework and acrylic resin teeth has been shown to have high success rates [86]. The strengths of this still popular solution are its simplicity, low cost, easy repair management, long tradition and clinician comfort level acquired over the years [87,88]. Alternative solutions, represented by metal–composite resin and metal–ceramic, are more expensive, more labor-intensive to fabricate, difficult to repair and subject to fabrication techniques [85,88,89]. Standard material ISFAFDPs are associated with several short- and long-term complications, including significant wear of the acrylic resin, fracture or delamination of the resin teeth, chipping and fracture of the ceramic veneer in functional areas, lack of passive fit, difficulty in achieving gingival pink coloration and costly prosthetic repairs [85,88,90,91]. The presence and length of cantilevers, lack of fine proprioception, type of antagonist, parafunctional overload and poor adhesion of ceramic veneers are the main risk factors associated with complications of these rehabilitations. Higher rates of prosthetic complications occur when ISFAFDPs are antagonized with natural teeth or fixed restorations [13]. Specifically, metal–acrylic resin ISFAFDPs require five to six maintenance procedures in 10 years, with higher numbers in cases of bimaxillary rehabilitation [88,90]. To overcome the limitations of traditional materials, the development of CAD-CAM technology has allowed for the introduction of zirconia–ceramic systems for screw-retained implant–prosthetic frameworks [92]. In the following years, advances in zirconia-based materials in terms of aesthetics and the need to resolve technical complications mainly related to chipping of the ceramic veneer led to the introduction of monolithic zirconia frameworks [85]. ISFAFDPs in monolithic zirconia represent a promising solution that not only demonstrates high biocompatibility and encouraging reliability data in the short and medium terms but also leads to a reduction in the complexity of prosthetic design, offering undeniable fabrication advantages [93]. There is currently no scientific evidence to support a link between the type of occlusal material and implant osseointegration. The choice of occlusal material appears to be irrelevant in terms of force transmission to the implants [94]. Furthermore, there appears to be no difference between occlusal materials in terms of stresses transmitted to the bone [95]. For a favorable long-term prognosis of ISFAFDPs, regular occlusal checks are recommended to ensure contact stability and proper load distribution [38,96]. In this sense, the choice of occlusal material leads to important differences in the management of the implant-supported restoration over time. In particular, resin, composite, glass–ceramic and zirconia occlusal surfaces experience different levels of occlusal wear with function and also exert different levels of abrasive wear on the antagonist (Table 3) [97]. Therefore, it is imperative to consider the wear properties of restorative materials and the presence of parafunctions when making decisions about prosthetic materials. Based on the previous considerations of occlusion and occlusal morphology, occlusal materials with high wear rates, such as acrylic resin, may not be suitable for definitive ISFAFDPs, as they would render all considerations of occlusal patterns, masticatory efficiency and occlusal stability moot.

### 3.6. Personalized Occlusion and Biomechanical Risk

Despite the popularity of implant dentistry, there is still a low level of scientific evidence on how to manage occlusion in cases of ISFAFDP, and the issue remains challenging for clinicians. Currently, decisions are made based on expert recommendations and clinical experience. Current approaches are based on those developed for removable complete dentures and fixed dentures on natural teeth. Many authors apply a logical and practical approach to achieve the primary goal of occlusal therapy in ISFAFDPs: comfort, masticatory function and stability over time [14,17]. Some authors believe that the choice of occlusal scheme has little clinical relevance, as most patients are able to adapt to changes [96]. Occlusal patterns also appear to play a relative role in mastication [14]. Regardless of the occlusal scheme adopted, the masticatory efficiency of implant-supported restorations is considered to be very close to that of the natural dentition, and the maximum masticatory force is as high, if not higher [98]. In contrast, occlusal patterns assume a major role in the development of functional forces that are transmitted to the supporting bone through prosthetic connections [14]. In ISFAFDPs, the reduction in proprioceptive feedback, combined with prosthetic stiffness, requires special attention to biomechanical factors [96]. Occlusion is not just simple contact between opposing surfaces but involves multiple inclined planes and different force vectors. Occlusal forces are multidirectional; complex; unpredictable; transient; and, often, non-intuitive [99]. Each occlusion/disocclusion cycle is characterized by transient loads that are very distant from traditional unidirectional force assumptions [99]. For these reasons, outlining truly biomechanically effective occlusal theories is very complex. The differences between teeth and implants underlie the occlusal theories applied in implant prosthetics. Some authors state that clinical success over time of implants can be achieved through biomechanically controlled occlusion [27]. The mechanically inspired occlusal scheme aims to reduce unfavorable occlusal forces in an attempt to prevent biomechanical complications. It envisions centralizing forces predominantly along the long axis of the implant body and minimizing horizontal forces and cuspal interference. An example of these occlusal theories is implant-protected occlusion (IPO) [100]. Currently, the lack of scientific evidence does not allow for the definition of the best occlusal scheme for ISFAFDPs. Each case must be evaluated individually and requires individualized planning [27]. The principle of ‘personalized occlusion’ of ISFAFDPs is based on the consideration of several individual factors that can condition the loads on the prostheses and implants: geometry, number, length, diameter, angulation and position of the implants; prosthetic type and design: prosthetic material; direction and magnitude of loads; status of the antagonist arch; jaw conformation; bone quality; and age and sex of the patient [14,27]. Different levels of force, in terms of magnitude, duration, type and direction, are applied in different patient conditions. Before analyzing occlusal patterns and morphology, it should be noted that axial loading is purely theoretical and that, in nature, occlusal forces on teeth are non-axial, and all implant-supported restorations are subject to non-axial loads. Although some occlusal theories consider the directionality of occlusal forces to be a critical factor, in ISFAFDPs, the recommendation to load implants primarily axially is a questionable goal and difficult to achieve. Since occlusal forces can exert high stresses on the framework and be transmitted to the implants and surrounding bone, in ISFAFDPs, the framework acts as a rigid implant splint, allowing for the distribution of occlusal contacts within a polygon determined by the implant arrangement and regulating the distribution of stresses from the framework to the implants and bone [101]. Contemporary “all-on-four” treatment with high success rates demonstrates the absence of detrimental effects of non-axial loading: distal tilted implants show better stress distribution than vertical distal implants, which show increased levels of peri-implant stress in the presence of cantilevers [79]. Regardless of the implant placement, passive framework adaptation is a non-negotiable requirement for ISFAFDPs: one of the main causes of static stress on the peri-implant bone in the absence of occlusal loading is the lack of passive fit between the implant framework and the abutments [51]. However, there is no scientific evidence of a cause-and-effect link between passive framework failure and biological effects on peri-implant bone [51]. On the basis of these considerations, we propose a biomechanical risk score index dedicated to ISFAFDPs: the aim is to provide the clinician with a practical and immediate tool to personalize prosthetic treatment and to allow for the implementation of design measures aimed at a favorable long-term rehabilitation prognosis (Table 4).

### 3.7. Clinical Recommendations on the Management of Occlusion in ISFAFDPs

In the literature, the occlusal concepts applied in ISFAFDPs refer to three reference schemes: mutually protected occlusion, group function and bilaterally balanced occlusion. The choice of occlusal scheme is defined according to the antagonist arch [11]. In cases of antagonistic implant rehabilitation with removable complete dentures, bilaterally balanced occlusion is indicated for better force distribution and better prosthetic stability [27]. Although studies on the subject are still scarce, there is a consensus that bilaterally balanced occlusion is also advantageous in terms of stability in cases of antagonistic arch rehabilitation with implant-supported overdentures [102]. Mutually protected occlusion and group function are the most commonly used occlusal schemes for ISFAFDPs antagonistic to natural dentition or fixed prosthetic rehabilitations. The mutually protected occlusion (canine guidance) scheme implies that during lateral excursions and protrusion, the posterior teeth are protected by the anterior guidance, whereas during centric occlusion, the anterior teeth are expected to be in light contact and protected by the posterior teeth. The alternative is a group function occlusion (also called unilateral balanced occlusion). It is definitely indicated in cases where the canines of the prosthetic or antagonist arch are to be protected from excessive excursion loads. Some authors consider a group function to be more advantageous in terms of load distribution [103]. It seems to promote patient comfort and reduce the mechanical stress on the prostheses during function [27,96]. A recent FEA study on “all-on-four” ISFAFDPs with different occlusal schemes, in addition to confirming higher stress values at the implant neck and peri-implant cortical bone, showed that the stresses would be more favorably distributed with group function [104]. Regardless of the occlusal scheme, the occlusion should provide bilateral stability in centric occlusion with equally distributed contacts and occlusal loads [102]. In addition, the occlusion should provide for a freedom in centric of approximately 1–1.5 mm. The concept of ‘freedom in centric’ was first introduced by Schuyler in the 1960s [105]. Dawson later coined the term ‘long centric’ to express the same concept [106]. A long centric occlusion requires the elimination of any occlusal interference between the maximum intercuspation (MIP) and the centric relation (CR). Freedom in centric allows for the avoidance of precontact during function [107] and promotes patient comfort, especially in cases of rehabilitated edentulous patients who have lost proprioception [96]. Some studies suggest providing non-exaggerated anterior guidance: the steeper the anterior guidance, the greater the horizontal forces on prostheses and anterior implants. Occlusal forces should be distributed as evenly as possible between the incisors from the centric to the edge-to-edge position. The extent of anterior overbite in addition to anterior guidance is conditioned by the esthetic result for which a slight vertical overlap of the anterior teeth is essential [96]. Regarding occlusal morphology in ISFAFDPs, occlusal strategies such as cusp inclination reduction, ‘shallow’ occlusal anatomy, and 10–20% reduced occlusal table born to minimize lateral forces and bending moments in implant-supported partial restorations [32] seem to assume less significance. In addition, the design of occlusal surfaces should consider that chewing efficiency is affected by changes in tooth size and shape [108]. Specifically, a reduction in occlusal surface area results in a decrease in the efficiency of food particle comminution per unit of masticatory work. A greater occlusal area has been correlated with increased masticatory efficiency in experimental studies [108]. In addition, the occlusal morphology of artificial teeth may influence masticatory function. A recent systematic review shows that anatomical teeth improve chewing efficiency and muscle activity in removable partial denture wearers. In contrast, non-anatomical teeth increase muscle activity, negatively affecting chewing [109]. These data suggest that the adoption of anatomical teeth with a not narrowed occlusal table represents a potentially more advantageous strategy than occlusal modifications, which may compromise chewing efficiency and force the patient to increase chewing cycles with a counterproductive effect in terms of occlusal loads (Table 5).

## 4. Conclusions

Biophysiological differences between teeth and dental implants and the issue of occlusal overload, although controversial, form the basis of occlusion management in ISFAFDPs. Occlusion management lacks scientific evidence: recommendations and suggestions on occlusion are mainly based on expert opinion and intuitive guidelines, without a high level of scientific evidence. However, it is clear that the favorable prognosis of ISFAFDPs is linked to a correct understanding of the biomechanical principles involved. In the design of ISFAFDPs, the lack of proprioceptive feedback requires special attention to biomechanical factors: minimizing overload complications and providing biomechanical stability are among the main goals of occlusion. Despite the lack of scientific evidence, occlusal overload can be considered a potential accelerating factor for peri-implant bone loss in the presence of plaque and inflammation. Moreover, overload is a recognized factor of mechanical complications in ISFAFDPs. Cantilever and bruxism represent the most critical biomechanical stressors and require personalized strategies aimed at defusing unfavorable forces. High-wear occlusal materials are incompatible with stable occlusion, proper load distribution and masticatory efficiency. In ISFAFDPs, the occlusion must be decided on the basis of several factors that can influence the loads on prostheses and implants: each case must be evaluated individually and requires a personalized occlusion. The biomechanical risk score index is designed to be a useful and immediate tool to personalize prosthetic treatment, allowing the clinician to implement arrangements aimed at a favorable long-term prognosis of ISFAFDPs. The choice of occlusal scheme should be defined on the basis of the antagonist arch according to criteria of advantageous load distribution.

## Figures and Tables

**Table 1:** Differences between teeth and dental implants.

Comparing Teeth and Dental Implants:
Teeth are equipped with the periodontal ligament; osseonitegrated implants are anchored to the bone, without the periodontal ligament apparatus. Occlusal thickness perception, teeth around 20 µm; implants from 48 to over 100 µm. Movement under load, teeth: axial movement 25–100 μm and horizontal movement 50–100 μm; implants: axial movement 3–5 μm and horizontal movement 10–50 μm. Teeth have periodontal mechanoreceptors and proprioceptive mechanisms to protect against occlusal overload; implants, without periodontal ligament mechanoreceptors and associated proprioceptive mechanisms, are susceptible to occlusal overload. Several studies support the existence of a compensatory phenomenon known as osseoperception, which is the mechanism underlying the functional integration of the dental implant: failure to achieve optimal osseoperception may expose it to overload.

**Table 2:** Clinical recommendations for avoiding occlusal overload on implants.

How to Avoid Occlusal Overload on Dental Implant Restorations:
Loading should be as axial as possible Reduced inclination of cusps Shallow occlusal anatomy and narrowed occlusal table (10–20% less) Wide freedom (1–1.5 mm) for maximum intercuspation and centric relation Limited extension of cantilever lengths Group function on the implant prosthesis if canines not present or periodontally compromised Anterior guidance independent of the presence of teeth or implants in the anterior area In case of severe bone resorption, cross-bite occlusion to reduce the buccal cantilever and to improve axial loading Clearance of around 50 µm between implants and opposing teeth (single-tooth implant restoration and Kennedy Class III, IV implant-supported fixed partial dentures) Select the restorative material with less wear property Periodic assessment and occlusal adjustments to prevent potential overloading due to positional changes and wears

**Table 3:** Wear of occlusal materials.

Wear of Occlusal CAD/CAM Materials After 5 Years of Clinical Service Antagonistic to Enamel
Acrylic polymer-based (PMMA) materials: wear of approximately 100 μm Composite: wear of approximately 40 μm Leucite glass ceramic: wear of approximately 40 μm Lithium disilicate: wear of approximately 33 μm Feldspathic ceramics: wear of approximately 23 μm Zirconia: wear of 0 μm

**Table 4:** ISFAFDP personalized risk factor score index.

Risk Factors	Score
Presence of parafunction, bruxism	1
Presence of cantilever	1
Cantilever length more than 10 mm (equivalent to 1 molar)	0.5
Cantilever A-P spread unfavorable (recommended 1.5:1)	1
Lower number of implants than recommended (6–8 implants in the upper jaw and 4–8 mandibular implants)	1
Presence of narrow diameter and/or short/ultrashort implants	0.5
Implants placed in poor quality bone for heavy load bearing area	0.5
Sub-optimal passive fit of prosthetic framework	0.5
Occlusal materials susceptible to wear	0.5
Double ISFAFDPs in antagonism	0.5
No occlusal splints in cases of parafunctions and/or bruxism	0.5
Predictive signs of biomechanical complications	
Wear of occlusal material	0.5
Fracture, chipping of occlusal material	0.5
Loosening of abutment/prosthetic screws	0.5
Fracture of abutment/prosthetic screws	1
≤2: Low biomechanical risk	
3–5: Moderate biomechanical risk	
>5: High biomechanical risk	

**Table 5:** Clinical recommendations on the management of occlusion in ISFAFDPs.

Occlusal Recommendations in ISFAFDPs:
Mutually protected occlusion or group function in the case of an antagonistic arch with natural dentition or with fixed prosthodontics or implant-fixed prostheses. Bilaterally balanced occlusion if ISFAFDP is antagonistic with a removable complete denture or overdenture. Bilateral stability in centric occlusion. Equally distributed contacts and occlusal loads. Simultaneous bilateral contact on canines and posterior teeth and light contact on incisors. Freedom in centric 1–1.5 mm (long centric). Anatomical teeth and occlusal table not narrowed. Minimal anterior overbite. Reduced anterior guidance in protrusive movements. In lateral movements, canine guidance or group function with less steep paths. Posterior cantilevers with clearance of approx. 100 μm and no contact in lateral and protrusive excursions. Cantilever extension not exceeding 12 mm in the upper jaw and 15 mm mandibular. In bruxer, short cantilevers (8 mm) and occlusal splints are recommended in selected cases. In cases of antagonism with a removable complete denture: during excursion movements, look for one or more balancing contacts and plan for more anteroposterior space for the front teeth. It is recommended to leave the most distal tooth slightly out occluded if posterior cantilevers are present. Occlusion design in definitive ISFAFDP is incompatible with high-wear occlusal materials.
Occlusal recommendations in immediate loading:
Avoiding cantilevers or minimizing the length of cantilevers. In lateral movements, group function or canine guidance with flat paths and minimal vertical overlap. In protrusive movements, guidance distributed over all anterior teeth, including canines, with flat paths and minimal vertical overlap. Although ISFAFDP is antagonistic to a removable complete denture, balancing contacts should be avoided in excursion movements at the expense of prosthesis instability.

## Data Availability

No data are available.
